# Supplementary material for: Expression of Toll-like receptors in oral squamous cell carcinoma
Source: PLoS One. 2024 Apr 9;19(4):e0300437. doi: 10.1371/journal.pone.0300437 (PMC11003673; doi:10.1371/journal.pone.0300437)
Supplement: Attachment — Submitted filename: Response to Reviewers.docx [file pone.0300437.s003.docx]

Dear Editor,

The authors thank you very much for considering this manuscript titled ‘Expression of Toll-like Receptors In Oral Squamous Cell Carcinoma’.

The manuscript has now been revised according to suggestions and comments from the reviewers.

We hope the article is now ready for publication.

Editor comment 1

As suggested by the reviewers authors must enrolled more number samples to justify their findings.

Author response:

Unfortunately, the sample size could not be achieved in higher number because new patients could not be enrolled due to the limited amount of tissue available.

**Reviewer 1 comment**

With this small number of sample size it is difficult to have a conclusion and statistical power. So authors have to increase number of samples to achieve logical conclusion.

Author response:

The authors highly appreciate the suggestion given and acknowledges the low number of sample size as one of the limitations of the study. However, the sample size could not be achieved in higher number at this point due to the limited amount of tissue available.

The authors have made changes to the abstract and text in the manuscript to reflect the limitation of small sample size.

In the manuscript the changes have been made as (in page number 2 and line number 48):

According to the limited number of samples, our data indicates downregulation of TLRs and NF-κB in OSCC, and upregulation of TLR4 expression with presence of *Candida*.

In the manuscript the changes have been made as (in page number 14, 15 and line number 449):

OSCC is a multifactorial disease linked with various risk factors and no single factor has been recognized as having independent influence on the prognosis of OSCC. During the progression of oncogenesis from a normal healthy cell to a pre-malignant or a potentially malignant cell, mutations in several DNA molecules occur leading to the loss of control of the growth of cell which eventually lead to uncontrolled proliferation of the cells leading to the cancer. To determine the genetic mechanisms involved in OSCC, the mutations should ideally also be analyzed using genetic techniques such as qPCR in order to measure mRNA levels in the tissue sample. Only immunohistochemical staining was used to compare the level of expression of TLR and NF-kB between the OSCC and HC samples in this study and is one of the limitations of this study. However, qPCR techniques do not provide information about the specific site within the tissue sample from where the signal is originated. While, in this study we have analyzed the immunolocalization and staining intensity of TLR1-10 and NF-κB in the different epithelial layers, basement membrane (BM) zone, endothelium, and infiltrative zone of tissue sections. In addition, a limitation of our study is the low number of sample size due to the limited amount of tissue available.

**Reviewer 2 comment**

 Authors have answered most of the comments without further experimentation. Still the number of samples is a concern.

Author response:

The authors highly appreciate the suggestion given and acknowledges the low number of sample size as one of the limitations of the study. However, the sample size could not be achieved in higher number at this point due to the limited amount of tissue available.

The authors have made changes to the abstract and text in the manuscript to reflect the limitation of small sample size.

In the manuscript the changes have been made as (in page number 2 and line number 48):

According to the limited number of samples, our data indicates downregulation of TLRs and NF-κB in OSCC, and upregulation of TLR4 expression with presence of *Candida*.

In the manuscript the changes have been made as (in page number 14, 15 and line number 449):

OSCC is a multifactorial disease linked with various risk factors and no single factor has been recognized as having independent influence on the prognosis of OSCC. During the progression of oncogenesis from a normal healthy cell to a pre-malignant or a potentially malignant cell, mutations in several DNA molecules occur leading to the loss of control of the growth of cell which eventually lead to uncontrolled proliferation of the cells leading to the cancer. To determine the genetic mechanisms involved in OSCC, the mutations should ideally also be analyzed using genetic techniques such as qPCR in order to measure mRNA levels in the tissue sample. Only immunohistochemical staining was used to compare the level of expression of TLR and NF-kB between the OSCC and HC samples in this study and is one of the limitations of this study. However, qPCR techniques do not provide information about the specific site within the tissue sample from where the signal is originated. While, in this study we have analyzed the immunolocalization and staining intensity of TLR1-10 and NF-κB in the different epithelial layers, basement membrane (BM) zone, endothelium, and infiltrative zone of tissue sections. In addition, a limitation of our study is the low number of sample size due to the limited amount of tissue available.
